# The *ropAe* gene encodes a porin‐like protein involved in copper transit in *Rhizobium etli* CFN42

**DOI:** 10.1002/mbo3.573

**Published:** 2017-12-27

**Authors:** Antonio González‐Sánchez, Ciro A. Cubillas, Fabiola Miranda, Araceli Dávalos, Alejandro García‐de los Santos

**Affiliations:** ^1^ Programa de Ingeniería Genómica Centro de Ciencias Genómicas Universidad Nacional Autónoma de México Cuernavaca Morelos México; ^2^ Deparment of Developmental Biology Washington University School of Medicine St. Louis MO USA; ^3^ Deparment of Civil and Environmental Engineering Massachusetts Institute of Technology Cambridge MA USA

**Keywords:** copper homeostasis, copper uptake, porins, *Rhizobium*, RopA

## Abstract

Copper (Cu) is an essential micronutrient for all aerobic forms of life. Its oxidation states (Cu^+^/Cu^2+^) make this metal an important cofactor of enzymes catalyzing redox reactions in essential biological processes. In gram‐negative bacteria, Cu uptake is an unexplored component of a finely regulated trafficking network, mediated by protein–protein interactions that deliver Cu to target proteins and efflux surplus metal to avoid toxicity. *Rhizobium etli* CFN42 is a facultative symbiotic diazotroph that must ensure its appropriate Cu supply for living either free in the soil or as an intracellular symbiont of leguminous plants. In crop fields, rhizobia have to contend with copper‐based fungicides. A detailed deletion analysis of the pRet42e (505 kb) plasmid from an *R. etli* mutant with enhanced CuCl_2_ tolerance led us to the identification of the *ropAe* gene, predicted to encode an outer membrane protein (OMP) with a β–barrel channel structure that may be involved in Cu transport. In support of this hypothesis, the functional characterization of *ropAe* revealed that: (I) gene disruption increased copper tolerance of the mutant, and its complementation with the wild‐type gene restored its wild‐type copper sensitivity; (II) the *ropAe* gene maintains a low basal transcription level in copper overload, but is upregulated when copper is scarce; (III) disruption of *ropAe* in an *actP* (*copA*) mutant background, defective in copper efflux, partially reduced its copper sensitivity phenotype. Finally, BLASTP comparisons and a maximum likelihood phylogenetic analysis highlight the diversification of four RopA paralogs in members of the *Rhizobiaceae* family. Orthologs of RopAe are highly conserved in the *Rhizobiales* order, poorly conserved in other alpha proteobacteria and phylogenetically unrelated to characterized porins involved in Cu or Mn uptake.

## 1 INTRODUCTION

Copper (Cu) is an essential trace element for aerobic organisms of the three domains of life. Cu can exist as an oxidized cupric ion (Cu^2+^) or as reduced cuprous ion (Cu^+^). The capacity of copper to alternate between these two oxidation states, (Cu^+^/Cu^2+^), makes this metal the ideal cofactor of key enzymes catalyzing redox reactions in vital biological processes, such as respiration, free radical detoxification, the methane cycle, photosynthesis, the carbon cycle, and the nitrogen cycle (Festa & Thiele, 2011; Rubino & Franz, 2012). Cells have to maintain intracellular copper in trace concentration; otherwise Cu^+^ can displace iron from iron‐sulfur clusters of metalloproteins, resulting in their inappropriate structure and function. In addition, free iron and Cu^+^ may cause hydrogen peroxide to generate an increase at the concentration of hydroxyl radicals by a Fenton type reaction which can damage proteins, lipids, and nucleic acids (Macomber & Imlay, 2009).

As copper is essential for life on earth, it is important to understand how cells fulfill their copper requirements and how trace concentrations are maintained to avoid toxicity.

Although the eukaryotic copper uptake system is well‐documented (Boal & Rosenzweig, 2009), little information is available about how prokaryotic cells import this metal.

The role of outer membrane proteins (OMP) in Cu^+^/Cu^2+^ acquisition was first suggested by Lutkenhaus (1977) who reported the isolation of copper‐resistant mutants, in *Escherichia coli* B/r; these appeared with a frequency of 10^−5^ in minimal medium plates with 20 μmol/L CuSO_4_. This phenotype was associated with the absence of outer membrane protein b (OmpC according to Lugtenberg and Van Alphen's nomenclature published in 1983) (Lugtenberg & Van Alphen, 1983) determined by membrane preparation and gel electrophoresis. Fifteen years later, Lutkenhaus' hypothesis was contradicted by studies with well‐characterized *E. coli* B/r isogenic *OmpC* mutants which maintained the same level of copper and silver resistance as the parental strain (Bavoil, Nikaido, & von Meyenburg, 1977; Li, Nikaido, & Williams, 1997).

In *Mycobacterium smegmatis* the uptake of nutrients and beta‐lactam antibiotics is mediated by the MspA, MspB, MspC, and MspD porins (Danilchanka, Pavlenok, & Niederweis, 2008; Stephan et al., 2005). Their role in copper transport was experimentally assayed in *Mycobacterium smegmatis* porin‐deleted mutants which grew poorly under trace concentrations of CuSO_4_ and simultaneously increased their copper tolerance when exposed to CuSO_4_ overload (Speer et al., 2013).

The potential role of multiple porins in the uptake of copper has also been suggested by the transcriptional profiles of copper–adapted cells of *Pseudomonas aeruginosa*. This study revealed that at least eight genes coding for different putative porins were downregulated in copper‐adapted cells (Teitzel et al., 2006).

Our research group has been working on the characterization of the transportome of divalent cations in the facultative diazotroph *Rhizobium etli* CFN42 (Cubillas et al., 2013, 2014), an α‐proteobacterium belonging to the *Rhizobiales* order that can live as a saprophyte in the rhizosphere of *Phaseolus vulgaris* plants or as a nitrogen fixer in symbiosis with the roots of *P. vulgaris* plants (Segovia, Young, & Martínez‐Romero, 1993). Its 6.5 Mb genome is partitioned in one circular chromosome and six plasmids (Gonzalez et al., 2006). The contribution of the *R. etli* pRet42e plasmid to ion balance is due to the presence of NepA, a member of the cation diffusion facilitator family (CDF) required to deal with high nickel concentrations, and the P_1B_‐ATPase, ActP, a copper efflux pump that confers copper resistance (Landeta et al., 2011).

In this study we report that an isogenic mutant of *R. etli* CFN42, named CFNX185, which lacks 200 kb of its 505–kb plasmid pRet42e, is more tolerant to CuCl_2_ than the wild‐type strain. Further analysis of pRet42e led us to the identification and characterization of the *ropAe* gene, coding for a putative outer membrane protein (OMP) whose absence increased copper resistance. The enhanced copper resistance phenotype observed in a double *actP− ropAe*− mutant suggests that disruption of *ropAe* reduces the intracellular copper supply, alleviating the copper toxicity observed in the *actP* single mutant that is defective in its copper efflux. Bioinformatic predictions, genetic experiments and transcriptional analyses allowed us to propose that a basal transcription level of *ropAe* facilitates copper transport across the outer membrane. However, under copper limitation, *ropAe* increased its transcription level, suggesting that it may play an important role in ensuring copper supply when the bacterium faces copper scarcity. A phylogenetic analysis revealed that RopAe belongs to the Porin_2 (PF02530) family and that it is distant from MspA, MspB, and MspC porins in *M. smegmatis,* from OmpC of *E. coli* (Li et al., 1997), from MnoP (*blr0095*) of *Bradyrhizobium japonicum* (Hohle, Franck, Stacey, & O'Brian, 2011), and from the copper‐regulated porins of *Pseudomonas aeruginosa* belonging to the OprD family (Teitzel et al., 2006). Three homologs of RopAe were found encoded in the chromosome of *R. etli* CFN42. The four RopA porin‐like transporters belong to different monophyletic groups suggesting a functional divergence among them.

## 2 EXPERIMENTAL

### 2.1 Bacterial strains, media, and growth conditions

The characteristics of the bacterial strains and plasmids used in this study are listed in Table S1. Bacterial growth was started from glycerol stocks (20% and stored at −70°C) propagated in rich PY medium containing 5 g/L peptone, 3 g/L yeast extract, and 15 g/L agar. After sterilization, 10 ml/L 0.7 mol/L CaCl_2_ was added. Minimal medium (Mm) was prepared from three solutions (A, B, C) and sterilized separately. Solution A contained 1.620 g/L sodium succinate hexahydrate as a carbon source, 0.534 g/L NH_4_Cl as a nitrogen source, 0.219 g/L K_2_HPO_4_, and 0.1 g/L MgSO_4_. The pH of this solution was adjusted to 6.8 before sterilization in autoclave. Solution B contained filter sterilized 0.025 g/5 ml FeCl_3_·6H_2_O and solution C contained 0.7 mol/L CaCl_2_·2H_2_O (autoclaved). A quantity of 1 ml of B solution and 2 ml of C solution were added to 1 L of A solution.

Antibiotics for *R. etli* were added at the following concentrations (μg/ml): nalidixic acid, 20; streptomycin, 100; gentamicin, 15; and tetracycline, 3. For *E. coli* the antibiotic concentrations were (μg/ml): kanamycin, 30; gentamicin and tetracycline 10.

### 2.2 Metal sensitivity assay

Metal sensitivity was determined with a plate assay using square Petri dishes with a grid as follows: 50 mmol/L stock solutions of Cu, Ni, Co, Zn, and Cd, 500 mM Fe, and 45 mM chloride salts (Sigma‐Aldrich, St Louis, MO) were prepared in Milli‐Q water, filter sterilized and added at increasing concentrations to solid (1.5% wt/vol agar) Mm. The *R. etli* overnight cultures were adjusted to OD_620_ = 0.7, washed twice with 10 mM of MgSO_4_, serially diluted (10^−1^ – 10^−6^) and spotted (20 μl) on solid Mm supplemented with or without metal ions. Growth was recorded after 5 days of incubation at 30°C. The total inhibitory concentration of copper for wild‐type *R. etli* CFN42 was 20 μmol/L. The Minimal Inhibitory Concentrations (MICs) of metals able to reduce, in at least one log‐unit, the growth (CFU) were previously determined (Cubillas et al., 2013) as follows: 100 μmol/L Ni, 100 μmol/L Co, 200 μmol/L Zn, 100 μmol/L Cd, 15 μmol/L Cu, 2.5 mmol/L Fe, and 30 mmol/L Mn.

### 2.3 DNA manipulation

Cloning, restriction digest, ligation, transformation, Southern blotting, and hybridization were performed according to standard protocols (Sambrook, Fritsch, & Maniatis, 1989).

### 2.4 Generation of site‐specific deletions

Deletions of pRet42e plasmid were obtained using the Cre/*loxP* system as previously described (Landeta et al., 2011). Briefly, the region to be eliminated was flanked, in direct orientation, by *loxP* sites present in pVEX1311 plasmid (Ayres, Thomson, Merino, Balderes, & Figurski, 1993) and in pIC20R (Marsh, Erfle, & Wykes, 1984). These suicide vectors, containing a PCR fragment (≥300 bp) of DNA sequences flanking the region to be eliminated were introduced by conjugation into *R. etli* CFN42. Single crossover recombination mediates the plasmids' cointegration at the target sequences. The Cre recombinase, introduced by conjugation, mediated the in vivo recombination of both *loxP* sites and the excision of the target DNA. Plasmid deletions were validated by changes in their electrophoretic mobility using a modification of Eckhardt gel electrophoresis procedure (Hynes & McGregor, 1990) that consists of a gentle lysis, running the SDS solution backwards, followed by the loading of wells with cells and starting the lysis, running the SDS solution forwards. The second validation was done by the absence of amplicons, using the total genome of the deleted strain as PCR template.

### 2.5 Site‐directed vector integration mutagenesis and mutant complementation

A 400 bp internal fragment of the target genes were amplified by PCR (see Table S2 for primers) and cloned into pK18mob Km^r^ suicide vector (Schäfer et al., 1994). Plasmids were introduced into *R. etli* CFN42 by conjugation and mutants with vector integration by single‐crossover were selected as Km^r^ clones. Vector integration in the target gene was verified by Southern blot, using the internal 400 bp of the target gene, amplified by PCR as the probe.

To complement the phenotype of the *ropAe* mutant, PCR‐amplified fragments containing the complete *ropAe* gene or its *ropAch1, ropAch2,* and *ropAch3* paralogs were cloned into the broad–host‐range pBBRMCS‐5 plasmid (Kovach et al., 1995) or in its derived expression, pSRK vector (Khan, Gaines, Roop, & Farrand, 2008), and introduced into *ropAe* mutant by conjugation (Table S1).

### 2.6 Construction of a *ropAe*/*actP* double mutant

A 1.3 kb PCR‐amplified BamH1–XhoI fragment of the *actP* gene was cloned into pBluescript II SK(+) vector. The HindIII–HindIII *ΩSp* interposon (Fellay, Frey, & Krisch, 1987) was inserted into the sole HindIII restriction site of PCR‐amplified *actP,* located 900 bp upstream of the TAG stop codon. The BamH1–XhoI fragment containing the *actP*::*ΩSp* was subcloned into pJQ200 SK (Quandt & Hynes, 1993) and then introduced by conjugation into wild type *R. etli* CFN42 using *E. coli* S17‐1 (Simon, 1984) as donor. The wild‐type *actP* gene was replaced by the *actP*::*ΩSp* by double homologous recombination in most of the transcojugants grown in the presence of sucrose 12.5%. The gene replacement was verified by Southern blot using Redyprime kit for labeling with [α‐^32^P] the DNA fragment used as probe**.** To construct the double mutant *ropAe*::pK18mob/*actP*::*ΩSp;* the wild‐type *ropAe* was disrupted by site‐directed vector integration of the pK18mob plasmid in the *actP*::*ΩSp* mutant background as described above.

### 2.7 Cu‐dependent transcriptional response of *ropAe* measured by qRT‐PCR

To determine the effect of either copper excess or deficiency on *ropAe* expression, two different experiments were performed. For the copper excess condition, PY cultures (20 ml, OD_620 nm_ = 0.65–0.75) of wt *R. etli* were exposed for 30 min to 0.5 mmol/L CuCl_2_. For the copper deprivation treatment, wt *R. etli* was grown overnight in 20 ml of PY with or without 2 mmol/L of the membrane impermeable Cu^+^/Cu^2+^ chelator bathocuproine disulfonic acid disodium salt (BCDS, Sigma) (Ding, Xie, & Kang, 2011). These were used to extract the mRNA using the TriPure isolation reagent (Roche). The total RNA (DNA free, 1 μg) was reverse transcribed to cDNA using ReverAid H minus FirstStrand cDNA Synthesis (Fermentas). Quantitative real‐time PCR was performed on StepOnePlus (Applied Biosystems) using Maxima Syber Green/ROX qPCR master Mix (Fermentas) and 1 μg of cDNA as template. The *ropAe*,* actP* and *hisCd* genes were amplified by using the primers listed in Table S2. Their expression levels in the presence of CuCl_2_ or CuCl_2_ plus the BCDS Cu^+^/Cu^2+^ chelator were normalized to the expression level of housekeeping *hisCd* gene (Salazar et al., 2010). The data represent averages of four independent experiments with three technical replicates each. The fold change in gene expression was calculated using the ΔΔC_*T*_ method (Schmittgen & Livak, 2008).

### 2.8 Bioinformatic prediction of subcellular localization of RopAe

The localization of RopAe was analyzed with two different predictors of subcellular localization with high accuracy for OMP (Bhasin, Garg, & Raghava, 2005). The multimodular PSORT‐B (Gardy et al., 2003) examines the query sequence for the presence of 12 different characteristics, such as amino acid composition, similarity to proteins of known localization, signal peptide, alpha helices, motifs, etc. (http://www.psort.org/). The second predictor was CELLO (Yu, Lin, & Hwang, 2004) which uses a single analytical module, a support vector machine based on *n*‐peptide composition (http://cello.life.nctu.edu.tw/). The scores for the different subcellular localization analyses are shown in table S3. Both methods assigned high probability values for the outer membrane localization of RopAe: PSORT‐B, probability 9.3 of 10 as maximum score; CELLO, score 4.105 of 5 as maximum score.

### 2.9 Searching for RopAe homologs in protein databases and bacterial genomes

Protein Blast program (BlastP) at NCBI with default settings, was used to search databases or bacterial genomes for RopAe homologs. BlastP was also used to align two sequences and estimate identity (%), similarity (%), query cover (%), and E value.

### 2.10 Phylogenetic analysis of the Porin_2 family (PF02530)

According to Pfam, the Porin_2 (PF02530) family includes 263 OMP from alpha proteobacteria. To assess the number of rhizobial porins contained in this family, as well as their taxonomic distribution and diversity, the porins of members of the *Rhizobiales* order were downloaded, filtering from other α‐proteobacteria with the species distribution sequence search tool included in pfam 30.0. A total of 145 OMP homologs belonging to 35 species of *Rhizobiales*, as well as OmpC (a nonspecific porin) of *E. coli,* MnoP (a manganese transporter) of *B. japonicum* (Hohle et al., 2011), MspA, MspB, MspC, and MspD porins of *M. smegmatis* (Speer et al., 2013) comprised the data set (Table S4) and were aligned against the HMMER profile using hmmalign. The most conserved regions shared among the OMPs were obtained from the alignment of putative RopAe with the HMMER profile. The resultant data set, containing 127 OMPs (Table S4), was used to infer the evolutionary relationships among the rhizobial OMPs belonging to the Porin_2 family (PF02530) relative to the other characterized porins. Due to the low bootstrap values observed in the 127 sequences phylogeny (Figure S2), we decided to select the clades where the RopAe proteins and their closest homologs were located and we run the analysis again including the characterized proteins. The resulting phylogeny (Figure 6) has higher bootstrap values that support the conclusions. The ML‐phylogenetic analysis was performed under the LG+G+f model using amino acid alignment. The phylogeny was built using a parallel P threads‐based version of RAxML v8.2.4, because this software supports SSE3 vector instructions (Stamatakis, 2014). The ML‐search started with 100 random seed trees and the best tree was selected. Rapid bootstrapping was used to assess the branch support (Stamatakis, 2014). The number of necessary replicates was estimated at 100 using the extended majority rule criterion (Pattengale, Aberer, Swenson, Stamatakis, & Moret, 2011).

## 3 RESULTS

### 3.1 Characterization of an *R. etli* mutant with increased copper tolerance

**Figure 1:**
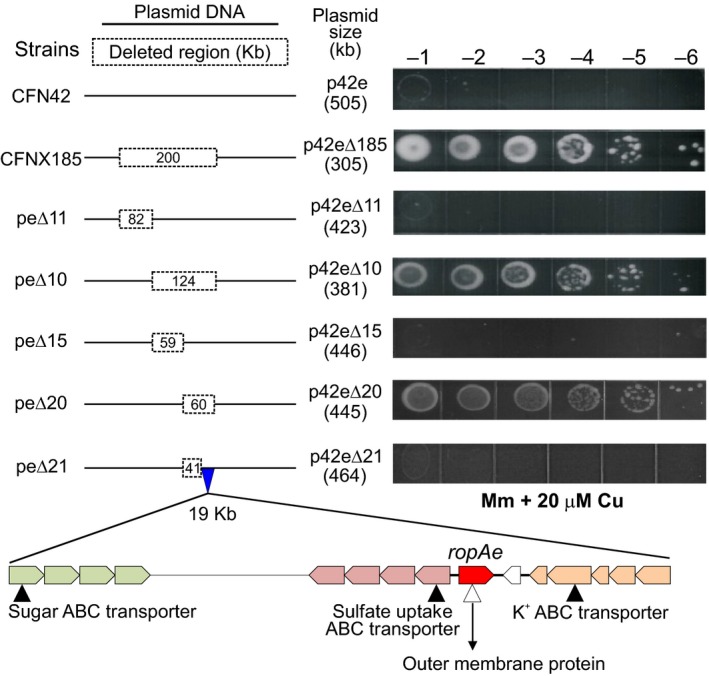
Identification of *ropAe* gene through the analysis of the copper‐resistance phenotype of wt *R. etli* CFN42 and its isogenic mutants harboring spontaneous (CFNX185) or engineered (Δ11 to Δ21) deletions of plasmid p42e (see Cu sensitivity assay in methods). *R. etli* CFN42 is unable to grow in minimal medium containing 20 μmol/L of CuCl_2_. Its isogenic mutant, *R. etli* CFNX185, lacking 200‐Kb of plasmid p42e, acquired the ability to grow in this medium. The copper‐resistance phenotype of mutants harboring sequential deletions of plasmid p42e (peΔ11 to peΔ21), covering the 200‐kb region lost in CFNX185, indicates that the absence of a 19‐kb fragment increases copper resistance. Four transporter‐coding genes contained in this fragment were disrupted by site‐directed vector integration (open and filled triangles). Only the mutation of *ropAe* (*RHE_PE00260*) increased Cu resistance (open triangle). Images are representative of five independent experiments

As a result of previous research conducted to analyze the functional contribution of the six *R. etli* CFN42 plasmids to contend with stress conditions, a collection of derivative mutants was constructed. The derivatives lack each of the following plasmids: pRet42a, pRet42b, pRet42c, pRet42d, and pRet42f, or a 200 Kb fragment of plasmid pRet42e (Brom et al., 1992). We assessed the toxic effect of Cu and other metals on these mutants by determining their growth capacity, as described in Materials and Methods, in minimal medium agar plates supplemented with increased concentrations of CuCl_2_ (0–25 μmol/L). The assays revealed that 20 μmol/L CuCl_2_ totally inhibited the growth of the parental strain (CFN42) (Figure 1) as well as that of the other plasmid‐cured strains (data not shown). In contrast, the derivative lacking 200 kb of pRet42e (strain CFNX185) showed full growth on 20 μmol/L CuCl_2_ (Figure 1). To identify the gene(s) responsible for the tolerance phenotype, consecutive small site‐directed deletions were constructed using the Cre/*loxP* system (see Materials and Methods) (Landeta et al., 2011). The increased copper tolerance observed in CFNX185 was also observed in strains with deletions peΔ10 (124 kb) and peΔ20 (60 kb); whereas deletion peΔ21 (41 kb) showed a wild‐type phenotype (Figure 1). This indicated that the gene(s) involved in copper resistance must be localized on a 19 kb region still present in plasmid peΔ21 (Figure 1, white triangle). The organization of the genes localized in this fragment and their predicted functions are listed in Table S5. A schematic representation of some of these genes is shown at the bottom of Figure 1, highlighting the presence of a 4,080 bp four‐gene cluster, *RHE_PE00245 to RHE_PE00248,* encoding a putative sugar ABC transport system, a sulphate uptake ABC transport system formed by five proteins *(RHE_PE00259 to RHE_PE00256*), and a putative OMP (*RHE_PE00260*, 1,017 bp) encoded close to a three‐gene cluster, which is part of the KdpA–KdpE (*RHE_PE00266‐RHE_PE00262*) potassium transporting system.

### 3.2 Disruption of *ropAe* increases copper tolerance in *R. etli* CFN42

**Figure 2:**
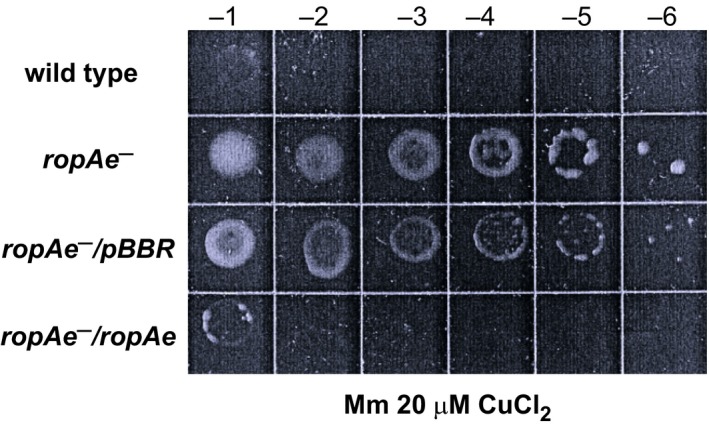
The disruption of *ropAe* increases copper resistance. The comparison of the growth presented by wt *R. etli* CFN42, the *ropAe* mutant (*ropAe−*), the mutant complemented with the empty vector (*ropAe−*/pBBR) and with the *ropAe* gene (*ropAe−*/*ropAe*) were assessed with the copper sensitivity plate assay described in the Methods section. Images are representative of five independent experiments

The analysis described above indicates that 10 out of 19 proteins encoded in the 19 Kb fragment of pRet42e might be involved in transport of small molecules and cations. To explain the copper resistance phenotype, we hypothesized that these proteins may participate in the uptake of divalent cations, including copper. Under a copper overload the absence of these Cu importers would reduce the transport of metal, resulting in a Cu resistance phenotype. To test this hypothesis, we constructed mutants in genes *RHE_PE00245* and *RHE_PE00259*, coding for solute binding proteins, annotated as components of the sugar and sulfate ABC transport systems, respectively; also in gene *RHE_PE00260,* coding for an uncharacterized OMP, and in *RHE_PE00263*, coding for a putative two‐component sensor histidine kinase, belonging to the putative potassium transport system (*kdpA*–*kdpE*) (Figure 1 and Table S5). The copper resistance phenotype of these mutants was assessed by determining growth of 10‐fold serial dilutions on minimal medium plates supplemented with 20 μmol/L CuCl_2_. The only mutation that produced an increased Cu resistance phenotype was that in the *RHE_PE00260* gene coding for a putative OMP labeled as *ropAe*. The complementation of the *ropAe* ^*−*^ mutant with the wild‐type gene, led to recovery of wild‐type levels of Cu sensitivity (Figure 2). These results indicate that the product of *ropAe* gene increases copper sensitivity.

### 3.3 Loss of *R. etli ropAe* gene does not enhance Mn^2+^, Cd^2+^, Ni^2+^, Fe^2+^, Co^2^, and Zn^2+^ resistance

According to the Transport Classification Data Base (Saier et al., 2016), most of the 16‐strand porins are classified as general or non‐specific transporters that allow the diffusion of hydrophilic substrates. To determine if RopAe mediates the sensibility to other divalent metal cations, *R. etli* and its isogenic *ropAe* mutant were grown on plates containing minimal inhibitory concentrations of Mn^2+^, Cd^2+^, Ni^2+^, Fe^2+^, Co^2+^, and Zn^2+^ (previously determined for the wild‐type strain). We found that the inhibitory effect of all these metals was similar for both strains (Figure S1).

### 3.4 The disruption of *ropAe* gene partially reverts the copper sensitivity phenotype of an *R. etli actP* mutant defective in copper detoxification

**Figure 3:**
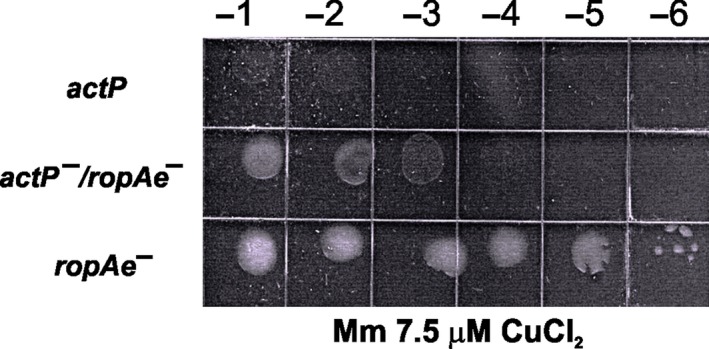
The disruption of *ropAe* counteracts the copper sensitivity of an *R. etli actP* mutant. This figure shows the copper resistance assessed as the growth ability of ten‐fold serially diluted cultures (OD_620 nm_ = 0.7) in Mm with 7.5 μmol/L CuCl_2_.The growth deficiency of an *actP* mutant is assumed to be due to a defective cytoplasmic detoxification of Cu^+^ by the inactivation of the P_1B_‐type ATPase encoded in *actP*. The partial growth recovery of the *actP−/ropAe−* double mutant may be due to a reduction in the influx of Cu^+^ by disruption of the *ropAe* gene. Images are representative of five independent experiments

In the genome sequence of *R. etli* CFN42, ORF *RHE_PE00007* is annotated as an *actP* homolog (*copA* in other bacteria), coding for a P_1B‐1_‐copper‐transporting ATPase protein, predicted to be located in the inner membrane, whose function is to pump copper out of the cytoplasm (http://genome.annotation.jp/rhizobase/Etli/genes/RHE_PE00007). Landeta et al. (2011) showed that *actP* disruption reduced the copper resistance of this bacterium, suggesting a deficiency in copper efflux. To get insights into the putative porin‐like role of *ropAe* in the uptake of copper, we hypothesized that an *actP*::ΩSp mutant, defective in cytoplasmic copper detoxification, should be rescued from a toxic copper overload (7.5 μmol/L CuCl_2_) if the putative entrance of copper is “closed” by mutation of the *ropAe* gene (*ropAe*::pK18 *mob* Km). As predicted, the *actP*::ΩSp/*ropAe*::pK18mob double mutant increased its copper tolerance in three orders of magnitude, compared to the single *actP* mutant (Figure 3). This result, together with the increased resistance to copper of the *ropAe* mutant, the Cu sensitivity of the complemented *ropAe* mutant, as well as the in silico structural analysis, reinforces the hypothesis that RopAe facilitates copper uptake.

### 3.5 The transcription of *ropAe* is upregulated under copper limiting conditions

To determine the effect of copper on the transcription of *ropAe*, its expression was analyzed by qRT‐PCR in the exponential growth phase (OD_620 nm_ = 0.7) of cultures exposed or unexposed for 30 min to a non‐inhibitory overload of 0.5 mmol/L CuCl_2._ These induction conditions were validated by measuring the copper‐inducible *actP* gene of *R. etli* CFN42 that encodes a putative P_1B‐1_‐Cu^+^‐ATPase efflux pump (Landeta et al., 2011).

**Figure 4:**
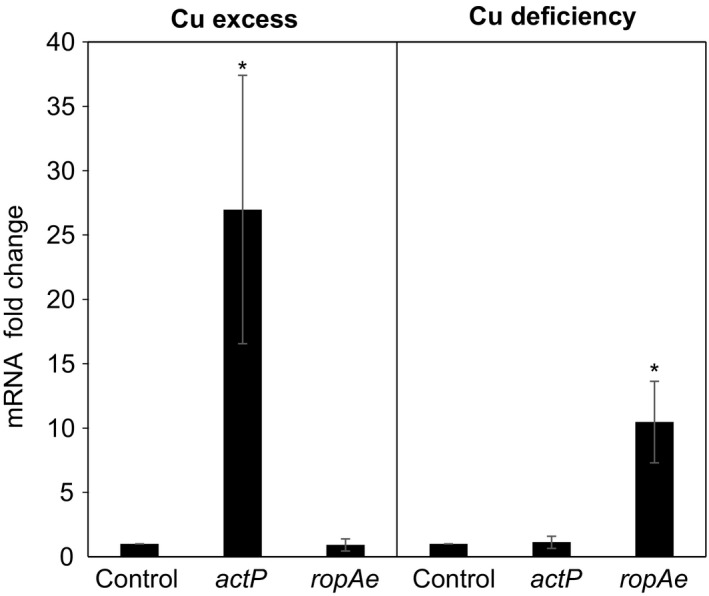
The expression of *ropAe* gene is upregulated under copper deficiency. The transcriptional responses of *actP* and *ropAe* to copper excess (PY + 0.5 mM CuCl_2_) and copper deficiency (PY+ 2mmol/L chelating agent BCDS) were analyzed by qPCR. The copper excess panel shows a 25‐fold increase in the mRNA level of *actP* when wt *R. etli* was exposed for 30 min to an overload of 0.5 mmol/L CuCl_2_. The copper deficiency panel shows a 17‐fold increase in the mRNA level of *ropAe* when *R. etli* was culturedovernight in the presence of 2 mmol/L of the Cu^+^/Cu^2+^chelating agent bathocuproine disulfonic acid (BCDS). Both assays were normalized to *hisCd* mRNA level (mean ± *SD*., *n *=* *3) (*t* test, **p ≤* *.05)

From studies in other bacterial models, such as *Agrobacterium fabrum* C58 (formerly *A. tumefaciens* C58), a close relative of *R. etli*, we assumed that the transcription of *R. etli actP* gene is activated by the monovalent copper‐dependent protein CueR that binds to a regulatory region of *actP* and induces its transcription through a conformational DNA change required for transcriptional activation of *actP* (Bittner, Kraus, Schäkermann, & Narberhaus, 2017; Nawapan et al., 2009). As expected, the relative expression of the *actP* gene increased 25‐fold after copper treatment (Figure 4). In contrast, no significant difference was found in the expression of *ropAe* in cultures treated similarly (Figure 4).

There are several examples cited in the literature of upregulation of high‐affinity porin‐encoding genes at lowmetal concentrations (Hohle & O'Brian, 2009; Patzer & Hantke, 1998; Speer et al., 2013). To determine whether *ropAe* is induced under copper limitation, its transcription was assessed in overnight cultures of wt *R. etli* supplemented with 2 mM of the Cu^+^/Cu^2+^ chelator agent bathocuproine disulfonic acid (BCDS). BCDS is a charged and membrane impermeable chelator commonly used in studies that require extracellular copper depletion (Asahi et al., 2014; Campos, Guzmán, López‐Fernández, & Casado, 2009; Ding et al., 2011; Labbé, Zhu, & Thiele, 1997). As can be seen in Figure 4b, the transcription of *ropAe* increased 10‐fold in cultures containing BCDS. The decrease in Cu in overnight cultures was validated by the reduction in *actP* expression in the presence of BCDS (Figure 4b).

### 3.6 The *ropAe* gene encodes a putative OMP that shares structural characteristics with porins

According to the annotation of the *R. etli* CFN42 genome, *ropAe* is an ORF of 1,017 nucleotides, located in plasmid p42e, encoding a 338 amino acid protein annotated as an uncharacterized porin predicted to be located in the outer membrane. This predicted location was reinforced using other bioinformatic tools. First, a BlastP search with default settings indicates that the overall sequence of RopAe is a domain shared with members of the Porin_2 family (porins from the α class of Proteobacteria) and that the OM_ channels a superfamily containg 16–18 beta‐stranded barrels. To support its outer membrane localization, RopAe was analyzed with two different predictors of subcellular localization (see Methods section for details) with high accuracy for OMPs (Bhasin et al., 2005; Table S3). The multimodular PSORT‐B (Gardy et al., 2003) and the CELLO predictor (Yu et al., 2004) are based on *n*‐peptide composition (http://cello.life.nctu.edu.tw/). Both methods assigned high probability values to the outer membrane localization of RopAe: the PSORT‐B probability was 9.3 of 10 as maximum score; the CELLO probability was 4.105 of 5 as maximum score (Table S3).

The closest homologs are putative OMPs mainly encoded in plasmids of other *R. etli* and *R. leguminosarum* strains, with identities ranging from 96 to 100% with a query coverage ≥95% (Table S6). The identity of RopAe to homologs present in other members of *Rhizobiales* fell in a range from 30% to 50%. To learn more about the structure of RopAe and its closest homologs, their amino acid sequences were analyzed with the transmembrane beta‐barrel topology prediction BOCTOPUS2 server (Hayat & Elofsson, 2012) (http://boctopus.bioinfo.se/). All of the analyzed putative OMP porins shared 16 transmembrane β‐strands distributed fromamino acids 58 to 338, which form part of the typical β‐barrel structure of porins (Table S6). We used RopAe homologs as queries against the CATH/Gene 3D (v4.1) protein domains database to predict the 3D structure adopted by these putative OMPs (Lam et al., 2016; Sillitoe et al., 2015) (http://www.cathdb.info/). This search revealed that all tested RopAe homologs, including the partially characterized OMP from *R. leguminosarum* 248 (de Maagd et al., 1992), share a porin domain of 266 amino acids present in the outer membrane proteins grouped in the OMP IIIA family. CATH/Gene 3D (v4.1) predicts “transport” as the associated biological process and “outer membrane” as the cellular component (Table S6). We also identified an additional putative porin present in multiple species of *Mesorhizobium* (NCBI Taxid 3744) that share 31% identity with RopAe. However, CATH/Gene 3D assigned these mesorhizobial porins to the OmpP2 family (Table S6).

### 3.7 The plasmid‐borne RopAe protein shares structural characteristics with three RopA homologs encoded in the chromosome of *R. etli* CFN42

As the *R. etli* genome is characterized by a high level of gene redundancy (Flores et al., 1987) we searched for RopAe homologs through a BLASTP search using RopAe as query against the proteome of *R. etli* CFN42. This search retrieved three chromosomally encoded RopAe homologs: RHE_CH01349 (RopAch1), RHE_CH02437 (RopAch2) and RHE_CHO3578 (RopAch3) which share 59% and 58% identities respectively with RopAe (Table S7). Although the four putative porins share structural characteristics such as the 16 transmembrane β strands and the predicted 3D model (Table S7), the increase in copper resistance of *ropAe* mutant could not be complemented with the chromosomally encoded *ropA* genes present in *cis*. To get insight into the functional role of RopAch1, RopAch2, and RopAch3 we attempted to obtain mutants by vector integration; however, Southern blot analyses revealed multiple fragments of unexpected sizes suggesting that these mutants contain more than one vector integration (data not shown). This is supported by a pairwise nucleotide sequence alignment that revealed an 80% to 90% identity shared among the three chromosomally encoded *ropA* genes (Table S8).

**Figure 5:**
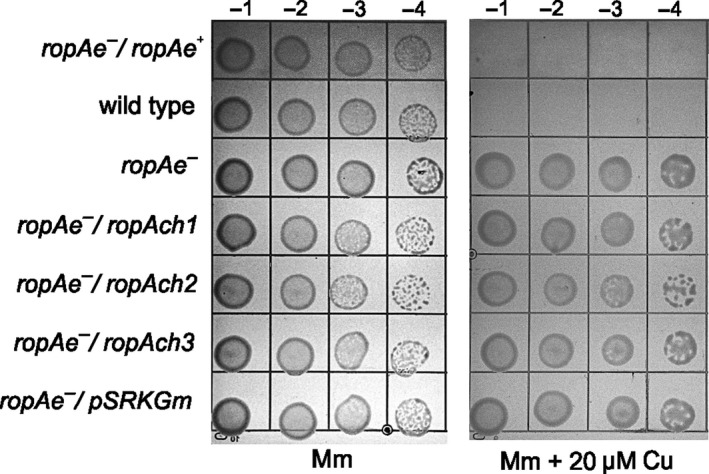
The enhanced copper‐resistance phenotype of *R. etli* CFN42 *ropAe* mutant (*ropAe* ^*−*^) could not be reduced by genetic complementation with the paralogs genes *ropAch1, ropAch2*, and *ropAch3*. The *ropAe* ^*−*^ mutants harboring each one of the paralogs genes were designated *ropAe* ^*−*^/*ropAch1, ropAe* ^*−*^/*ropAch2,* and *ropAe* ^*−*^/*ropAch3*. As controls we used the *ropAe* ^*−*^ mutant complemented with the *ropAe* wild type (*ropAe* ^*−*^/ropAe) and *ropAe* ^*−*^ mutant complemented with the empty vector (*ropAe* ^*−*^/pSRKGm). The resistance or sensitivity phenotypes were assessed by growth in the presence or absence of 20 μmol/L Cu with the copper sensitivity plate assay described in the experimental section. Images are representative of three independent experiments

Alternatively, the functional redundancy of these genes in copper transport was investigated using a genetic complementation approach. The three chromosomally‐encoded *ropAch* genes and *ropAe* were cloned into the pSRKGm vector and introduced into the *R. etli* mutant *ropAe*::pK18mob by conjugation. The transconjugants harboring *ropAch1*,* ropAch2* or *ropAch3* maintained the same copper resistance level as the mutant *ropAe*::pK18mob. In contrast, the transconjugant *ropAe* gene was able to reduce the enhanced copper resistance of the *ropAe* mutant. These data support the hypothesis that RopAe and RopAchs play different roles (Figure 5).

Furthermore, the location of the four *ropA* homologs in different gene contex (Table S7) suggests that these genes could be following different evolutionary pathways, as has been observed in genes from other models analyzed with different bioinformatic gene contex tools (Dewey, 2011; Martinez‐Guerrero, Ciria, Abreu‐Goodger, Moreno‐Hagelsieb, & Merino, 2008; Puggioni et al., 2014; Seret & Baret, 2011). In support of the functional divergence hypothesis and outer membrane protein localization, it was recently reported that two *R. etli* RopAe paralogs, RopAch1 and RopAch2, were found to be expressed in the exoproteome of this organism when exposed to the plant flavonoid naringenin (Meneses et al., 2017).

### 3.8 RopAe defines a new class of porins distantly related to those involved in Mn and Cu uptake

The BlastP search in the nonredundant database for *R. etli* RopAe homologs also revealed the presence of a conserved domain that **s**pans from amino acid 33 to 322 (E value 1.93^−72^) and groups these proteins in the Porin_2 (PF02530) Pfam. This family clusters OMPs from α‐proteobacteria that share this domain. The compilation of these data revealed that the presence of multiple RopA porins is a widespread characteristic in the genomes of members of the *Rhizobiales*. The number of *ropA* per genome varies from one to nine, with three being the most frequent number of *ropA* per genome (Table S4).

**Figure 6:**
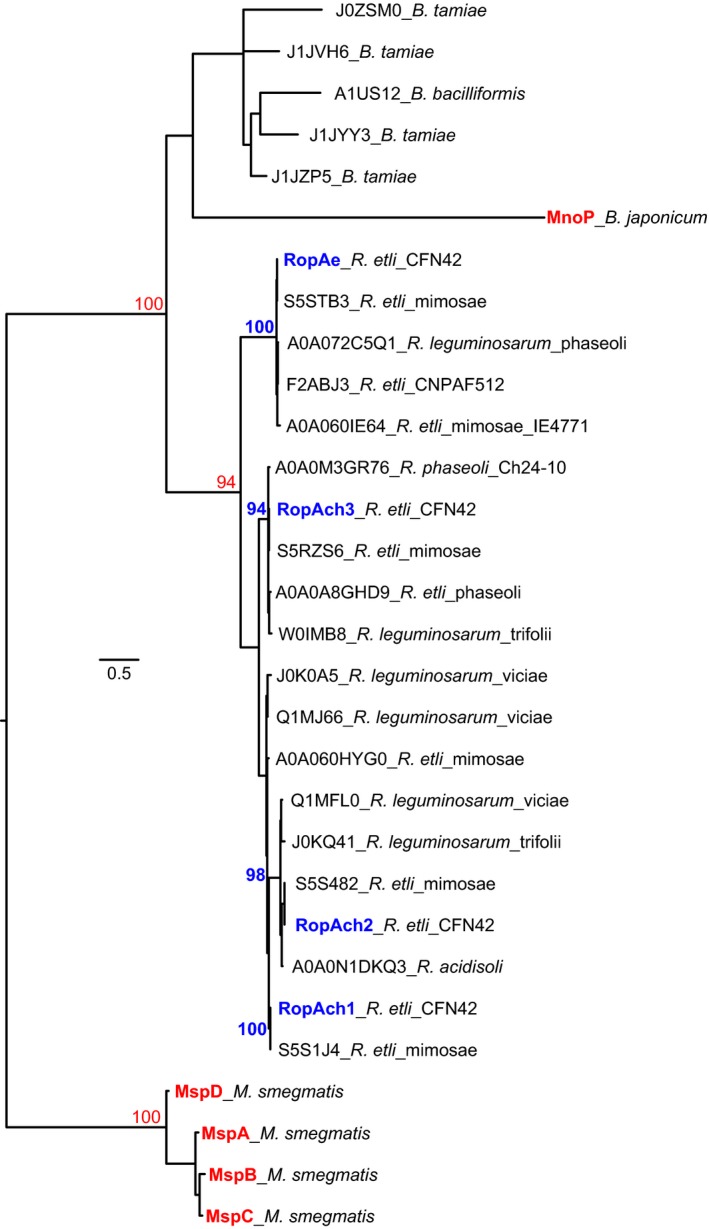
The putative RopA OMPs of rhizobia define a new family of porins phylogenetically unrelated to already characterized metal uptake porins. A maximum likelihood phylogenetic tree inferred from a subset of 30 proteins analyzed in a larger phylogeny (Figure S2) showed that RopA proteins clearly define a monophyletic clade of proteins distantly related to characterized metal uptake proteins MnoP (Mn) and MspA‐D (Cu), supported by bootstrap values ≥90. The diversification of *R. etli* and *R. leguminosarum* RopAe and its homologs RopAch, is evinced by its separation into two clades. The copper porins of *M. smegmatis*, the general porin OmpC of *E. coli* and the high‐affinity Mn^2+^ importer MnoP of *B. japonicum* used as outgroups, are distantly related to *R. etli* porins (Figure S2). The scale bar indicates the expected number of amino acid substitutions per site under the LG + G  +  f model.

To understand the evolutionary relationships of RopAe and paralogs in relation to other OMPs involved in metal trafficking, we aligned the rhizobial porins grouped in the Porin_2 (PF02530) family, characterized porins involved in Mn (MnoP from *B. japonicum*) and Cu (MspA−D from *M. smegmatis*) uptake and the best‐studied porin of γ‐proteobacteria, OmpC. The *P. aeruginosa* OprC, OprD, OprE, OprG, and OprN porins, reported to be downregulated in Cu‐adapted culture, were excluded from the alignment because they have an e‐value higher than the used cut‐off (<1e^−3^) (Table S8). The maximum likelihood‐based phylogeny inferred from the alignment of 127 amino acid sequences of rhizobial porins was used to define major groups of proteins (Figure S2) including non‐RopA protein homologs found in rhizobia. A phylogeny containing the closest RopA homologs as well as multiple out‐group characterized proteins (Figure 6) clearly shows that rhizobial RopAe homologs belong to a new class of porins distantly related to those known to be involved in Mn and Cu trafficking.

## 4 DISCUSSION

As studies on copper uptake are extremely scarce in prokaryotes, the identification and characterization of proteins involved in the acquisition of this metal are a topic of major interest in the study of copper homeostasis in bacteria. The best characterized bacterial proteins involved in copper acquisition are the MspA porin and its paralogs in *M. smegmatis* (Speer et al., 2013). In gram‐negative bacteria there is only a study of putative porins which are downregulated in copper‐adapted cultures of *P. aeruginosa* (Teitzel et al., 2006).

As part of a research project focused on understanding copper trafficking in the facultative diazotroph *R. etli* CFN42, we searched for mutants with enhanced copper resistance in a collection of *R*. *etli* mutants that have lost whole plasmids or fragments of them, both spontaneously or through programmed deletions. Using this approach we identified a plasmid‐encoded gene, annotated as *ropAe,* whose disruption enhanced copper resistance, and we further determined that the wt sensitivity could be recovered upon introduction of the wt *ropAe* gene. According to the annotation of the genome at NCBI, the *ropAe* gene (*RHE_PE00260*) is located in plasmid p42e and encodes a protein of 338 amino acids, identified as a porin outer membrane protein. The presence of a putative domain containing a multi‐pass transmembrane protein that forms a pore of passive diffusion across the outer membrane is predicted between amino acids 33 and 332. Based on this information we hypothesized that *ropAe* encodes a putative OMP involved in copper uptake. In this study we performed bioinformatics, genetics and gene expression analyses to examine this hypothesis.

The OM localization was supported by an in silico analysis of the amino acid sequence using two different predictors with high accuracy for OMPs: PSORT‐B and CELLO. Both predictors assigned high probability values for OM localization. The structural characteristics of RopAe, such as the transmembrane beta‐barrel topology and the 3D structure adopted by OMPs were well supported using BOCTOPUS2 server and CATH/gene 3D.

We hypothesized that if *ropAe* encodes a porin‐like protein that contributes to the normal supply of copper, we would expect that a *ropAe* mutant will show alterations in copper‐dependent functions due to a reduction in copper availability. In support of this proposal we found that an *R. etli* CFN42 *actP* mutant, defective in cytoplasmic detoxification of copper due to a Tn*5* disruption of its P_1B1_‐Cu‐transporting ATPase, cannot grow in the presence of 7.5 μmol/L CuCl_2_. However, the *actP/ropAe* double mutant partially recovered its growth ability in 7.5 μmol/L CuCl_2._ The results of our experiments suggest that this is due to a deficiency in copper supplementation produced by the disruption of *ropAe*. These data need to be corroborated by determining the transport activity of RopAe.

The inhibitory effect of other divalent metal cations was similar for both strains (Figure S1). This result suggests that RopAe does not facilitate the uptake of metals other than copper, or alternatively, that RopAe might mediate a defective uptake for these metals that does not result in toxic levels in the cytoplasm. Otherwise, there could be a functional redundancy among different porins for these metals, as reported for *M. smegmatis* (Speer et al., 2013).

We also determined that the transcription of *ropAe* did not change when *R. etli* was exposed to a CuCl_2_ overload. As expected, the expression of *actP*, coding for a copper efflux pump, increased 25‐fold after exposure to the same copper overload. In contrast, under copper‐limiting conditions, the transcription of *ropAe* is upregulated and the expression of *actP* is turned off. Low copper conditions were established with the chelator agent BCDS, a charged and membrane impermeable chelator commonly used in studies that require an extracellular copper depletion condition. The BCDS chelates Cu^+^ but is also known to bind Cu^2+^, forcing a tetrahedral chelator geometry that facilitates the reduction in Cu^2+^ to Cu^+^ (Asahi et al., 2014; Campos et al., 2009; Ding et al., 2011; Labbé et al., 1997). The BLASTP search was restricted to the genome of *R. etli* CFN42 using its own *ropAe* as query; it revealed the presence of three *ropAe* homologs located in distant regions of the *R. etli* CFN42 chromosome. As none of these homologs substitutes for *ropAe,* we proposed a functional divergence between RopAe and its chromosomally encoded homologs RopAch1, RopAch2, and RopAch3, which is also supported by the phylogenetic analysis that separates the plasmid and chromosomally encoded RopA homologs into two different clades.

A complex evolutionary story of porin‐encoding genes is assumed by the presence of gene redundancy in both prokaryotic and mitochondrial genomes. The presence of multiple copies may have an important role in the stress response of the cell, maintaining a function in the case of loss of primary genes (Bay, Hafez, Young, & Court, 2012; Pinne et al., 2006; Saccone et al., 2003). For instance, in *M. smegmatis*, the porin‐mediated copper uptake requires the cooperation of redundant porin homologs; a phenotype of increased Cu resistance could only be obtained in double and triple porin mutants (Speer et al., 2013).

All the data described above strongly support the hypothesis that *ropAe* encodes a porin‐like protein that facilitates copper diffusion by increasing its expression under low copper concentrations. Future experiments will address the transport properties of RopAe and its role, if any, in symbiosis with *Phaseolus vulgaris* plants in the presence of copper.

## CONFLICT OF INTEREST

None declared

## Supporting information

**   **
