# Supplementary figures and images for: The ropAe gene encodes a porin‐like protein involved in copper transit in Rhizobium etli CFN42
Source: Microbiologyopen. 2017 Dec 27;7(3):e00573. doi: 10.1002/mbo3.573 (PMC6011978; doi:10.1002/mbo3.573)

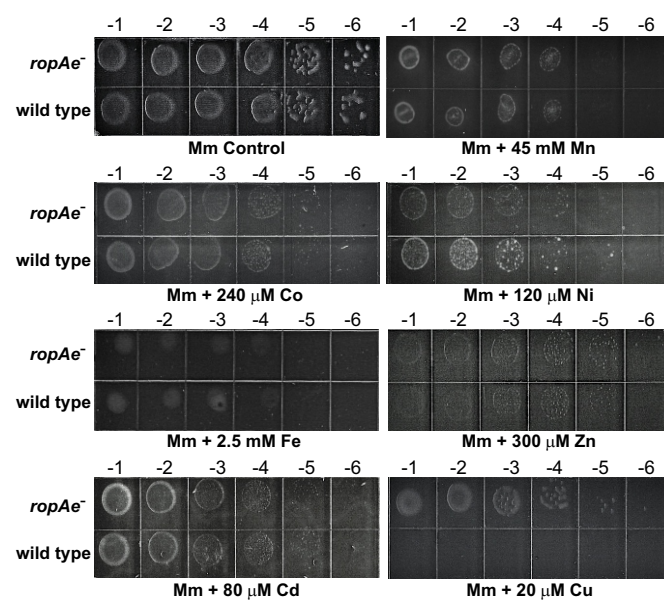

Supplement: Supplementary file 1 [file MBO3-7-e00573-s001.pdf]

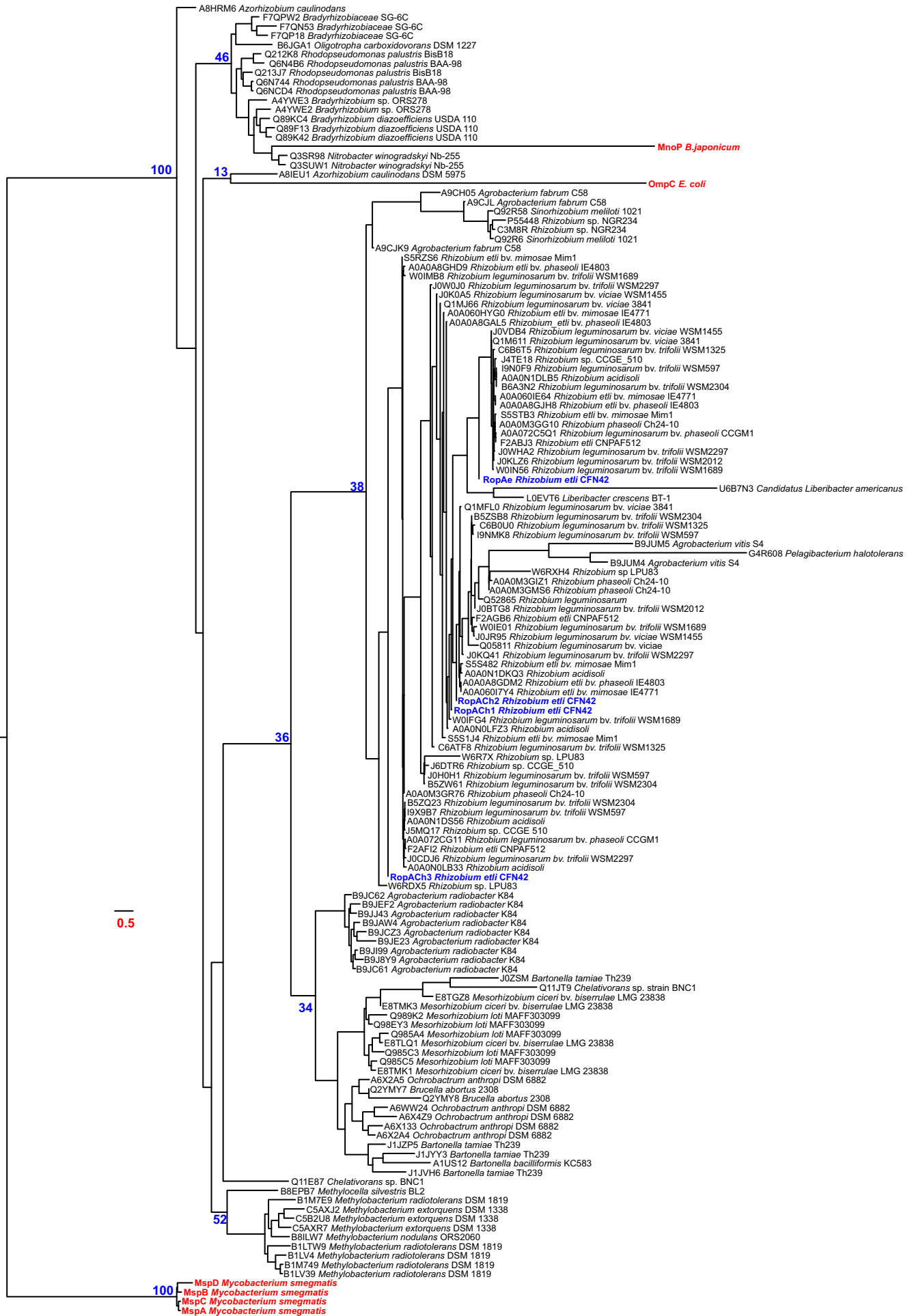

Supplement: Supplementary file 2 [file MBO3-7-e00573-s002.pdf]
