# Supplementary material for: The ropAe gene encodes a porin‐like protein involved in copper transit in Rhizobium etli CFN42
Source: Microbiologyopen. 2017 Dec 27;7(3):e00573. doi: 10.1002/mbo3.573 (PMC6011978; doi:10.1002/mbo3.573)
Supplement: Supplementary file 3 [file MBO3-7-e00573-s003.pdf]

Table S1. Bacterial strains and plasmids used in this study

Rhizobium etli strains	Relevant Genotype	References
CFN42	Wild type, Nal^r, p42e 505 kb	Segovia et al. 1993
CFNX185	CFN42 p42e lacking 210.097 kb (from 87,541 to 297, 638)	Brom et al. 1992
peΔ11	CFN42 p42eΔ11, lacking 82.028 kb (from 91,132 to 173,160)	Landeta et al. 2011
peΔ10	CFN42 p42eΔ10, lacking 124.160 kb (from 173,900 to 298,060)	Landeta et al. 2011
peΔ15	CFN42 p42eΔ15, lacking 59 kb (from 174,447 to 233,534)	This study
peΔ20	CFN42 p42eΔ20, lacking 60 kb (from 233,531 to 294,302)	This study
peΔ21	CFN42 p42eΔ21, lacking 41.194 kb (from 233,531 to 274,725)	This study
PE00245 Km^r	CFN42 *RHE_PE00245* ::pK18mob Km^r	This study
PE00259 Km^r	CFN42 *RHE_PE00259* ::pK18mob Km^r	This study
CFN42 *ropAe*$^-$	CFN42 *ropAe* ::pK18mob Km^r	This study
CFN42 *kdpD*$^-$	CFN42 *kdpD* ::pK18mob Km^r	This study
CFN42 *actP*$^-$	CFN42 *actP* ::ΩSp^r	This study
CFN42 *ropAe*$^-$ */ropAe*	CFN42 *ropAe* ::pK18mob Km^r complemented with *ropAe* wild type	This study
	cloned into pBBR1MCS5 Gm^r	This study
CFN42 *ropAe*$^-$ *actP*$^-$	CFN42 *ropAe* ::pK18mob Km^r actP::ΩSp^r	This study
CFN42 ropAe-/ropAe	CFN42 *ropAe* ::pK18mob/complemented with *ropAe* into pSRK Gm^r	This study
CFN42 *ropAe -/ropAch1*	CFN42 *ropAe* ::pK18mob/complemented with *ropAch* 1 into pSRK Gm^r	This study
CFN42 *ropAe -/ropAch* 2	CFN42 *ropAe* ::pK18mob/complemented with *ropAch* 2 into pSRK Gm^r	This study
CFN42 *ropAe -/ropAch* 3	CFN42 *ropAe* ::pK18mob/complemented with *ropAch3* into pSRK Gm^r	This study
***E. coli* strains and plasmids**	**Relevant Genotype**	**Reference**
DH5α	host for recombinant plasmids, Nal^r	Stratagene
S17-1	Donor for conjugation	Simon 1984
DH5α/pK18 mob Km^r	pK18 suicide vector, mob, Km^r used for gene disruption	Schäfer et al. 1994
DH5α/pK18mob/*ropAe*	788 bp EcoRI-HindIII fragment of *ropAe* cloned into pK18mob Km^r	This study
DH5α/pK18mob/*PE00245*	463 bp EcoRI-XbaI frag. of *PE00245* gene cloned into pK18mob Km^r	This study
DH5α/pK18mob/*PE00249*	354 bp SmaI-HindIII frag. of *PE00249* gene cloned into pK18 mob Km^r	This study

DH5α/pK18mob/*PE00259*	581 bp EcoRI-HindIII frag. of *PE00259* gene cloned into pK18mob Km^r	This study
DH5α/pK18mob/*kdpD*	547 bp EcoRI-HindIII frag. of *kdpD gene* cloned into pK18mob Km^r	This study
DH5α/pBBR1MCS-5	Broad host range cloning vector, *mob* , Gm^r	Kovach et al. 1995
DH5α/pSRK Gm^r	pBBRMCS-5-derived expression vector lac promoter, *lacI* ^{q} , *lacZ* α^+ , Gm^r	Khan et al 2008
DH5α/pSRK Gm^r/*ropAe*	1,211 bp HindIII-KpnI amplicon containing *ropAe* into pSRK Gm^r	This study
DH5α/pSRK Gm^r/*ropAch1*	1,054 bp NdeI-BamHI amplicon containing ropAch1 into pSRK Gm^r	This study
DH5α/pSRK Gm^r/*ropAch2*	1,081 bp BamHI-KpnI amplicon containing *ropAch2* into pSRK Gm^r	This study
DH5α/pSRK Gm^r/*ropAch3*	1,104 bp BamHI-KpnI amplicon containing *ropAch3* into pSRK Gmr	This study

Table S1. Bacterial strains and plasmids used in this study (Continue)

***E. coli* strains and plasmids**	**Relevant Genotype**	**References**
DH5α/pBBR1MCS-5/*ropAe*	1,249 bp KpnI-XbaI containing *ropAe* gene in pBBR1MCS-5 Gm^r	This study
pVEX1311	VEX derivative with *loxP* , Spr	Ayres et al. 1993
pIC20RDA	pBR322 derivative *loxP* in BglII-ClaI, *oriT* (RK2) in XhoI, Tc^r in HindIII	Marsh et al. 1984
pBBCre	pBBRMCS5 with *cre* gene from pBS7	Ayres et al. 1993
PE00146 /pIC	1,028 bp BamHI-XbaI fragment of *PE00146* cloned into pIC20R	This study
PE00198 /pVEX 1311	884 bp BamHI-XbaI fragment of *PE00198* cloned into pVEX1311	This study
PE00199 /pIC	884 bp BamHI-XbaI fragment of *PE00199* cloned into pIC20R	This study
PE00265 /pVEX1311	1,700 bp BamHI-XbaI fragment of *PE00265* cloned into pVEX1311	This study
PE00199 /pVEX1311	884 bp BamHI-XbaI fragment of *PE00119* cloned into pVEX1311	This study
PE00244 /pIC20R	1,134 bp BamHI-XbaI fragment of *PE00244* cloned into pIC20R	This study

