# Supplementary material for: The ropAe gene encodes a porin‐like protein involved in copper transit in Rhizobium etli CFN42
Source: Microbiologyopen. 2017 Dec 27;7(3):e00573. doi: 10.1002/mbo3.573 (PMC6011978; doi:10.1002/mbo3.573)
Supplement: Supplementary file 4 [file MBO3-7-e00573-s004.pdf]

Table S2. Primers used in this study

Primer name	Sequence 5'-3' (restriction sites used for cloning are underlined)	Restriction sites used for cloning
ropAe-F-mutation	GCGACGGAATTCGGACCGGCTATTTTTA	EcoRI
ropAe-R-mutation	CGATAAGCTTGCCTGTTGTGTAGGCGCT	HindIII
ropAe-F-complementation	ACA GTT GGT ACC CGA AAG CGC CAT GGT G	KpnI
ropAe-R-complementation	GCC GAT CTA GAG GTG TAA CAA AGG CGC GG	XbaI
ropAe-F-qPCR	CGT ACC GAA GTG CGC TTC G	
ropAe-R-qPCR	GGG TGG TCT CGT TGC TGG	
hisCd-F-qPCR	ATC ATC GCA ACG CTA TCT CC	
hisCd-R-qPCR	CGA TGG CGA GAC AGC TAA AT	
actP-F-qPCR	CGA CGG CCA AAC ATT TCT TAA A	
actP-R-qPCR	TCG TCA GCG GGA GGA ATA	
RHE_PE00245-F-mutation	GGC ACG AAT TCC GAC ATC ATC TCG CTG C	EcoRI
RHE_PE00245-R-mutation	TGA AGT TCT AGA TGT CGC CGG CCA TCG T	XbaI
RHE_PE00249-F-mutation	ATT GGC CCG GGT TTA TCA AGT TCG GGG A	SmaI
RHE_PE00249-R-mutation	CGT CGA AGC TTA TCT TGT CGG GAG CCA G	HindIII
RHE_PE00259-F-mutation	TCA TCG GAA TTC CGG TCA TGG CAG GCA G	EcoRI
RHE_PE00259-R-mutation	GGC GCC AAG CTT GAG AAC AGG AAC ATG C	HindIII
RHE_PE00263-F-mutacion	AGG AGA GAA TTC CGC TCG TCG CCG GTT T	EcoRI

RHE_PE00263-R-mutacion	CAG GGG AAG CTT ATG GCA TGA GCC TGC A	HindIII
actP::Ω Sp F BamHI	CTG GAT CCC GCT TCC GTG CCC GTC TAT TTCG	BamHI
actP::Ω Sp R XhoI	CCG CTC GAG GCA AGG GCC GGC GCA TCG T	XhoI
ropAe-F-complemetation-ropAch1	CAT ATG TTG GAG GTC ATT TAT GA	NdeI
ropAe-R-complemetation-ropAch1	GGA TCC AGT CAG ACC AAA TTA	BamHI
ropAe-F-complemetation-ropAch2	GCC CCG GGA TCC AAA GGA ATG GAT TGG T	BamHI
ropAe-R-complemetation-ropAch2	CGA GGA GGT ACC ACA CTC ACC AAG TGG AT	KpnI
ropAe-F-complemetation-ropAch3	CGG TCT GGA TCC TCT CGC ATC TGA ACT G	BamHI
ropAe-R-complemetation-ropAch3	CGG TCT GGT ACC CAG CCT TGA TTA GAA C	KpnI
ropAe-F-complemetation-ropAe+	ACA GTT AAG CTT CGA AAG CGC CAT GGT G	HindIII
ropAe-R-complemetation-ropAe+	GCC GAG GTA CCG GTG TAA CAA AGG CGC GG	KpnI
