# Supplementary material for: The ropAe gene encodes a porin‐like protein involved in copper transit in Rhizobium etli CFN42
Source: Microbiologyopen. 2017 Dec 27;7(3):e00573. doi: 10.1002/mbo3.573 (PMC6011978; doi:10.1002/mbo3.573)
Supplement: Supplementary file 5 [file MBO3-7-e00573-s005.pdf]

Table S3. Predictions of subcellular localization of RopA proteins assessed by two different predictors.

Predictors											
	PSORTb v.3.0 (Probability value: 0 to 10)						CELLO v2.5 (Probability value: 0 to 5)				
	Subcellular localization[1]						Subcellular localization[1]				
**Query**	EC	**OM**	P	IM	C		EC	**OM**	P	IM	C
RopAe RHE_PE00260	0.03	**9.3**	0.03	0.01	0		0.561	**4.105**	0.066	0.061	0.206
RopAch1 RHE_CH01349	0	**10**	0	0	0		0.288	**4.52**	0.113	0.025	0.058
RopAch2 RHE_CH0247	0	**10**	0	0	0		0.155	**4.74**	0.049	0.021	0.030
RopAch3 RHE_CH078	0	**10**	0	0	0		0.288	**4.57**	0.071	0.031	0.035

[1]EC, extracellular; OM, outer membrane; P, periplasm; IM inner membrane; C, Cytoplasm.
