# Supplementary material for: The ropAe gene encodes a porin‐like protein involved in copper transit in Rhizobium etli CFN42
Source: Microbiologyopen. 2017 Dec 27;7(3):e00573. doi: 10.1002/mbo3.573 (PMC6011978; doi:10.1002/mbo3.573)
Supplement: Supplementary file 6 [file MBO3-7-e00573-s006.pdf]

Table S4. Phylogeny data set (Out group porins are yellow highlighted)

Strains	Porin ID	Total porins
Agrobacterium fabrum C58	A9CH05, A9CJK9, A9CJL0	3
Agrobacterium radiobacter K84	B9JC61, B9JI99, B9JC62, B9J8Y9, B9JAW4, B9JE23, B9JJ43, B9JCZ3, B9JEF2	9
Agrobacterium vitis S4	B9JUM4, B9JUM5	2
Azorhizobium caulinodans DSM 5975	A8IEU1, A8HRM6	2
Bradyrhiobium japonicum JCM 10833	Q89Y60	1
Bartonella bacilliformis KC583	A1US12	1
Bartonella tamiae Th239	J0ZSM0, J1JVH6, J1JZP5, J1JYY3, J0ZSM0	4
Bradyrhizobiaceae SG-6C	F7QPW2, F7QN53, F7QP18	3
Bradyrhizobium diazoefficiens USDA 110	Q89LZ7, Q89KC4, Q89F13, Q89K42	4
Bradyrhizobium sp. ORS278	A4YU63, A4YWE2, A4YWE3	3
Brucella abortus 2308	Q2YMY8, Q2YMY7	2
Candidatus Liberibacter americanus	U6B7N3	1
Chelativorans sp. BNC1	Q11E87, Q11JT9	2
Escherichia coli K12	P06996	1
Liberibacter crescens	L0EVT6	1
Mesorhizobium ciceri biovar biserrulae LMG 23838	E8TMK1, E8TLQ1, E8TMK3, E8TGZ8	4
Methylobacterium extorquens DSM 1338	C5AWY3, C5AXJ2, C5AXR7, C5B2U8	4
Methylobacterium nodulans	B8ILW7	1
Methylobacterium radiotolerans DSM 1819	B1M0H0, B1LV40, B1M7E9, B1LV39, B1M749, B1LTW9	6
Methylocella silvestris BL2	B8EPB7	1
Mycobacterium smegmatis ATCC 700084	A0QPU4, A0QR29, A0R3I3	3
Nitrobacter winogradskyi Nb-255	Q3SUW1, Q3SR98	2
Ochrobactrum anthropi DSM 6882	A6X2A5, A6X133, A6X2A4, A6WW24, A6X4Z9	4
Oligotropha carboxidovorans	B6JGA1	1
Pelagibacterium halotolerans	G4R608	1
Rhizobium etli CFN 42	Q2K0Q0, Q2K7H2, Q2KAI4, Q2K4A3	4
Mesohizobium loti MAFF303099	Q985C5, Q985C3, Q985A4, Q98EY3, Q989K2	5
Rhizobium sp. LPU83	W6RDX5, W6RXH4, W6R7X0	3

Rhizobium sp. NGR234	C3M8R2, C3M8R0, P55448	3
**Strains**	**Porin ID**	**Total porins**
Rhizobium_acidisoli	A0A0N0LB33, A0A0N0LFZ3, A0A0N1DKQ3, A0A0N1DLB5, A0A0N1DS56	5
Rhizobium_etli CNPAF512	F2ABJ3, F2AFI2, F2AGB6	3
Rhizobium_etli_bv_mimosae IE4771	A0A060HYG0, A0A060I7Y4, A0A060IE64	3
Rhizobium_etli_bv_mimosae Mim1	S5RZS6, S5S1J4, S5S482, S5STB3	4
Rhizobium_etli_bv_phaseoli IE4803	A0A0A8GAL5, A0A0A8GDM2, A0A0A8GHD9, A0A0A8GJH8	4
Rhizobium_leguminosarum	Q52865	1
Rhizobium_leguminosarum_bv_phaseoli CCGM1	A0A072C5Q1, A0A072CG11	2
Rhizobium_leguminosarum_bv_trifolii WSM2304	B5ZQ23, B5ZSB8, B5ZW61, B6A3N2	4
Rhizobium_leguminosarum_bv_trifolii WSM1325	C6ATF8, C6B0U0, C6B6T5	3
Rhizobium_leguminosarum_bv_trifolii WSM597	I9N0F9, I9NMK8, I9X9B7	3
Rhizobium_leguminosarum_bv_trifolii WSM2012	J0BTG8, J0KLZ6	2
Rhizobium_leguminosarum_bv_trifolii WSM597	J0H0H1	1
Rhizobium_leguminosarum_bv_trifolii WSM2297	J0KQ41, J0W0J0, J0WHA2, J0CDJ6	4
Rhizobium_leguminosarum_bv_trifolii WSM1689	W0IE01, W0IFG4, W0IMB8, W0IN56	4
Rhizobium_leguminosarum_bv_viciae WSM1455	J0JR95, J0K0A5, J0VDB4	3
Rhizobium_leguminosarum_bv_viciae	Q05811	1
Rhizobium_leguminosarum_bv_viciae 3841	Q1M611, Q1MFL0, Q1MJ66	3
Rhizobium_phaseoli Ch24-10	A0A0M3GG10, A0A0M3GIZ1, A0A0M3GMS6, A0A0M3GR76	4
Rhizobium_sp CCGE510	J4TE18, J5MQ17, J6DTR6	3
Rhodopseudomonas palustris BAA-98	Q6N744, Q6NCD4, Q6N4B6	3
Rhodopseudomonas palustris BisB18	Q212K8, Q213J7	2
Sinorhizobium meliloti 1021	Q92R60, Q92R58	2
Total porins		145
Total species: 38		
