# Supplementary material for: The ropAe gene encodes a porin‐like protein involved in copper transit in Rhizobium etli CFN42
Source: Microbiologyopen. 2017 Dec 27;7(3):e00573. doi: 10.1002/mbo3.573 (PMC6011978; doi:10.1002/mbo3.573)
Supplement: Supplementary file 7 [file MBO3-7-e00573-s007.pdf]

Table S5. Predicted function of 19 genes encoded on plasmid p42eΔ21 and the Cu tolerance phenotype of the mutants.							
**No.**	**ID**	**Gene symbol**	**Predicted gene product[1]**	**Nucleotides/Amino acids**	**Gene disruption[2]**	**Enhanced Cu resistance[3]**	
1	*RHE_PE00245*		sugar ABC transporter, sugar-binding protein	1260/419	YES	NO	
2	*RHE_PE00246*		sugar ABC transporter, permease protein	930/311	NO	nd[4]	
3	*RHE_PE00247*		sugar ABC transporter, permease protein	831/276	NO	nd	
4	*RHE_PE00248*		sugar ABC transporter, ATP-binding protein	1059/352	NO	nd	
5	*RHE_PE00249*		hypothetical protein	900/299	NO	nd	
6	*RHE_PE00250*		hypothetical protein	819/272	NO	nd	
7	*RHE_PE00251*		putative DNA ligase (ATP) protein	1065/354	NO	nd	
8	*RHE_PE00252*	*ligD*	ATP-dependent DNA ligase	2649/882	NO	nd	
9	*RHE_PE00253*		hypothetical protein	315/104	NO	nd	
10	*RHE_PE00254*	*pepT*	peptidase T	1233/410	NO	nd	
11	*RHE_PE00255*		putative cell wall-associated hydrolase protein	819/272	NO	nd	
12	RHE_PE00256		sulfate uptake ABC transporter, ATP-binding protein	1041/346	NO	nd	
13	RHE_PE00257		sulfate uptake ABC transporter, permease protein	873/290	NO	nd	
14	RHE_PE00258		sulfate uptake ABC transporter, permease protein	858/285	NO	nd	

No.	ID	Gene symbol	Gene product	Nucleotides/Amino acids	Gene disruption	Enhanced Cu resistance	
15	*RHE_PE00259*		sulfate uptake ABC transporter, substrate-binding protein	1026/341	YES	NO	
16	*RHE_PE00260*	*ropAe*	porin outer membrane protein	1017/338	YES	YES	
17	*RHE_PE00261*	*ptsNe*	enzyme II of the phosphotransferase system	510/169	NO	nd	
18	*RHE_PE00262*	*kdpE*	two-component response regulator	693/230	NO	nd	
19	*RHE_PE00263*	*kdpD*	two-component sensor histidine kinase	2709/902	YES	NO	
	[1]Data taken from RhizoBase (http://genome.microbedb.jp/rhizobase/Etli/genes/RHE_PE00245)						
	[2]Mutants without increased Cu resistance phenotype are highlighted in green						
	[3]Mutant with increased Cu resistance is highlighted in yellow						
	[4]nd: not determined						
