# Supplementary material for: The ropAe gene encodes a porin‐like protein involved in copper transit in Rhizobium etli CFN42
Source: Microbiologyopen. 2017 Dec 27;7(3):e00573. doi: 10.1002/mbo3.573 (PMC6011978; doi:10.1002/mbo3.573)
Supplement: Supplementary file 8 [file MBO3-7-e00573-s008.pdf]

Table S6. Structural characteristics of *R. etli* RopAe and its closest rhizobial orthologues.

Strain	Size (aa)	Uniprot ID	E value	% I[1]	% S[2]	Genome location	Sub cellular location (Psortb v 3.0.2)	Number of TM[3] β strands (BOCTOPUS 2)	Homology with 3D model CATH gene 3D	Porin Genomic context Potassium/ Sulphate ABC transport system4
R. etli CFN42 RopAe	338	Q2K0Q0	0	100	100	p42e	OM	16	OMP IIIA	YES[4]
R. etli bv. mimosae Mim1 RopA4	338	S5STB3	0	100	100	pRetMIM1d	OM	16	OMP IIIA	YES
R. etli CIAT652 RopAa	338	B3Q1J3	0	99	99	pA	OM	16	OMP IIIA	YES
R. leguminosarum bv viciae 3841 RopA3	338	Q1M611	0	96	98	pRL11	OM	16	OMP IIIA	YES
R. leguminosarum bv viciae WSM1325 Porin	338	C6B6T5	0	96	97	pR132502	OM	16	OMP IIIA	YES
R. phaseoli Ch24-10 RopAa	338	A0A0M3GG10	0	98	99	pRphch24	OM	16	OMP IIIA	YES
R. phaseoli Brasil 5 RopA3	338	A0A1W6GR24	0	98	99	prphabra5c	OM	16	OMP IIIA	YES
Rhizobium tropici CIAT899	366	L0LL14	4E-31	38	54	Chromosome	OM	16	Porin OmpP2b	NO[5]
Sinorhizobium mliloti 1021 RopA1	339	F7X0L1	4E-93	50	67	Chromosome	OM	16	OMP IIIA	NO
Mesorhizobium multiple species	392	NCBI Taxid 3744	1,00E-26	31	43	unavailable	OM	16	OmpP2	NO

[1]Percent identity

[2]Percent similarity

[3]TM, transmembrane β strand

[4]YES, means co-occurrence of RopA porin together with sulphate and potassium ABC transport system.

[5]NO, means absence of sulphate and potassium ABC transport systems in genome context
