# Supplementary material for: The ropAe gene encodes a porin‐like protein involved in copper transit in Rhizobium etli CFN42
Source: Microbiologyopen. 2017 Dec 27;7(3):e00573. doi: 10.1002/mbo3.573 (PMC6011978; doi:10.1002/mbo3.573)
Supplement: Supplementary file 9 [file MBO3-7-e00573-s009.pdf]

Table S7. Structural characteristics of plasmid-encoded RopAe and its three chromosomally encoded homologues RopACh

Rhizobium etli CFN42 **RopAe homologues**	**Uniprot ID**	**Length aa**	**% I/S[1]**	**Query Coverage %**	**TM β-strands[2]**	**CATH 3D model[3]**	**Genomic context[4]**
R. etl i CFN42 RopAe	Q2K0Q0	338	100/100	100	16	OMP IIIA	Potassium/Sulphate ABC transport system
R. etli CFN42 RopACh1	Q2KAI4	340	59/72	100	16	OMP IIIA	Cell wall and membrane biogenesis
R. etli CFN42 RopACh2	Q2K7H2	343	59/72	100	16	OMP IIIA	Guanine biosynthesis and lipids metabolism
R. etli CFN42 RopACh3	Q2K4A3	347	58/67	91	16	OMP IIIA	MFS transport system

[1]RopAe was used as query in BlastP 2 sequences searches. I, identity; S, similarity.

[2]Trans membrane β strands were searched in http://boctopus.bioinfo.se/

[3]The most probable 3D structure were searched in http://www.cathdb.info

[4]Genomic context was searched in microbial genomic context viewer at mcgv.embi.ru.nl
