# Supplementary material for: The ropAe gene encodes a porin‐like protein involved in copper transit in Rhizobium etli CFN42
Source: Microbiologyopen. 2017 Dec 27;7(3):e00573. doi: 10.1002/mbo3.573 (PMC6011978; doi:10.1002/mbo3.573)
Supplement: Supplementary file 10 [file MBO3-7-e00573-s010.pdf]

Table S8. Comparison of nucleotide sequences of *R. etli* putative porins.

Query sequence	Subject sequence	Identity (%)	Query cover (%)	E value
ropAch1	*ropAch2*	91	93	0.0
ropAch1	*ropAch3*	85	100	0.0
ropAch2	*ropAch3*	81	93	0.0
ropAe	*ropAch1*	68	96	$3e^{-92}$
ropAe	*ropAch2*	66	84	$6e^{-63}$
ropAe	*ropAch*	66	100	$2e^{-82}$
